# Women’s health service access and associated factors in Ethiopia: application of geographical information system and multilevel analysis

**DOI:** 10.1136/bmjhci-2022-100720

**Published:** 2023-04-28

**Authors:** Addisalem Workie Demsash, Agmasie Damtew Walle

**Affiliations:** Mettu University, College of Health Science, Health Informatics Department, Mettu, Ethiopia

## Abstract

**Objectives:**

Women’s access to healthcare services is challenged by various factors. This study aimed to assess women’s health service access and identify associated factors.

**Methods:**

A cross-sectional study design with a two-stage stratified sampling technique, and 12 945 women from the 2016 Ethiopia Demographic and Health Survey dataset were used. The spatial hotspot analysis and purely Bernoulli-based model scan statistics were used to highlight hot and cold spot areas, and to detect significant local clusters of women’s health service access. A multilevel logistic regression analysis was used to assess factors that affect women’s access to health services. A variable with a p<o.o5 was considered as a significant factor.

**Results:**

Overall, 29.8%% of women had health services access. 70.2% of women had problems with health services access such as: not wanting to go alone (42%), distance to health facilities (51%), getting the money needed for treatment (55%) and getting permission to go for medical care (32.3%). The spatial distribution of health service access in Ethiopia was clustered, and low health service access was observed in most areas of the country. Women who lived in primary, secondary and tertiary clusters were 96%, 39% and 72% more likely to access health services. Educational status, rich wealth status, media exposure and rural residence were statistically significant factors.

**Conclusions:**

In Ethiopia, women have problems with health services access. The spatial distribution of health services access was non-random, and hotspot areas of women’s health service access were visualised in parts of Benishangul Gumez, Amhara, Afar, DireDawa, Harari, and Somali regions. Creating job opportunities, public health promotion regarding maternal health service utilisation and constructing nearby health facilities are required for better healthcare service access for women.

**WHAT IS ALREADY KNOWN ON THIS TOPIC**

- Access to maternal healthcare services remains a significant problem in the world. Women’s health service access is poor in low-income and middle-income countries and maternal and child deaths are high.

**WHAT THIS STUDY ADDS**

- This study used nationally representative data. Application of geographical information system was applied, and women’s health service access was spatially presented and visualised through maps. Factors that affect women’s health service access were identified.

**HOW THIS STUDY MIGHT AFFECT RESEARCH, PRACTICE OR POLICY**

- The findings are crucial for stakeholders to give priority attention to the cold spot areas of women’s health service access, and create awareness for women regarding health service access. This study illustrates the practical application of geographical information systems to concerned women’s health service access, and the findings are used as a basis for future studies.

## Introduction

Globally, around 800 women died daily from preventable causes, pregnancy and childbirth.^1^ Worldwide, nearly 300 000 women were predicted to be died in 2010.^2^ Maternal and child mortality remains a major public health challenge in the developing world, and the discrepancy is high according to developed countries. The maternal mortality ratio in low-income and middle-income countries is 15 times higher than in developed countries.^3^ Sub-Saharan Africa had the highest maternal mortality ratio which is 64 deaths per 10 000 live births, and 900 maternal deaths occurred in Ethiopia.^3^ According to the 2011 Ethiopian Demographic and Health Survey (EDHS), maternal mortality rates were 6.76 per 1000 live births.^4^

Maternal and child mortality occur due to countries’ poor utilisation of healthcare services.^4^ An average of 52% of women received at least four antenatal care (ANC) services in developing regions. It was 36% in Asia, 49% in sub-Saharan Africa^5^ and 32% in Ethiopia.^6^ In addition, 77.69%, 73.95% and 67.61% of women delayed their first ANC visit in 2005, 2011 and 2016 EDHS, respectively.^7^ Pregnancy-induced high blood pressure, stillbirth and unsafe abortions are extremely high. Though institutional delivery is used to reduce pregnancy and birth risks, home birth in low-income countries is high and institutional delivery is 26%–32.5% in Ethiopia.^8^

Women’s postnatal care (PNC) service utilisation is insufficient, which is 6.9% with marked spatial heterogeneity in Ethiopia.^9^ Maternal and child health problems occur due to the inaccessibility of health institutions, poor women’s health-seeking behaviours, maternal and child health services inaccessibility, low media exposure, poor attitude and knowledge, and low-quality service provision.^8 10 11^ The geographical accessibility of healthcare services, rural women lack reliable transportation, and women are late in starting ANC service.^12^

In resource-limited settings, women’s access to health services is mainly affected by four principal factors such as not want to go alone, distance to health facilities, getting the money needed for treatment, and getting permission for medical care. Previous studies have proven that distance to a health facility (long distance) and the geographical position of health facilities,^13^ poverty, low monthly income and not having an occupation that makes women not have enough money for medical care,^12^ inadequate awareness and low-risk perception of women, laziness and disease severity that make women not want to go alone for medical care,^14^ and husbands’ and relatives’ complete decision-making culture for women’s health service access^15 16^ are challenges for maternal and child health service access among women.

Maternal and child healthcare services are the most effective and potential health interventions to overcome maternal and child mortality. Of these maternal and child healthcare services, the provision of ANC and PNC services,^17^ institutional delivery services,^18^ skilled birth assistance, and nutritional and breastfeeding counselling services are the main strategies of the Millennium Development Goal to reduce mortality and morbidity. Moreover, health facilities provide preventative, curative health services to prevent maternal and child deaths.^9^

Maternal health services would be accessible and fairly distributed,^19^ the quality of health service provision should be ensured^20^ and sufficient health professionals would available in health facilities. Plus, policy-makers understand women’s health services access problem and geographical variations of health service access to formulate strategies and interventions to solve women’s health services problems and to provide equity and quality of services provision.^17 21^ Therefore, this study would be an input for policy-makers to alleviate women’s health service access problems. Studies regarding women’s health service access are not adequate, and the findings of previous studies were insufficient and limited in spatial variation analysis of women’s health service access in Ethiopia. Moreover, the findings of this study would be important for women’s decision-making regarding maternal health service access, and the finding could support policy-makers, and programmers to design interventions for achieving women’s health service access. Therefore, this study aimed to assess women’s health services access, locate women’s health service access spatially and identify factors associated with women’s health service access.

## Methods

### Study design

A cross-sectional study design was used.

### Study setting

The study was conducted across nine regions of Ethiopia. Ethiopia has nine regional states with two city administrations. The country is located in the Horn of Africa and is bordered by Eritrea to the north, Djibouti, and Somalia to the east, Sudan and South Sudan to the west, and Kenya to the South.

### Data sources

The 2016 EDHS dataset was used. The 2016 EDHS was the fourth Demographic and Health Survey (DHS) conducted in Ethiopia. The survey was conducted by Ethiopian Public Health Institute at the request of the Federal Ministry of Health. According to the Ethiopian EDHS report, the survey was conducted from 18 January 2016 to 27 June 2016. The actual data for this study were accessed from the measure DHS website (www.dhsprogram.com), and Ethiopian shape files were downloaded from the open Africa website (https://africaopendata.org/dataset).

### Sampling producers

The 2016 EDHS was conducted in 2007 by the Central Statistical Agency. The census frame is the complete list of 84 915 enumeration areas that cover an average of 181 households. A two-stage stratified cluster sampling was used, each region was stratified into urban and rural areas. At the first stage of selection, a total of 645 enumeration areas were selected independently with probability proportion to each enumeration areas. In second stage of selection, a fixed number of 28 households per cluster were selected with an equal probability of systematic selection from the newly created household listing. For more detail about the methods, visit the 2016 EDHS report.^22^

### Study populations

All women who were either permanent residents of the selected households or visitors who stayed in the household the night before the survey were the source population. Whereas, all women aged 15–49 years were the study population. Zero coordinates areas, clusters that had no defined proportions of health service access and irregularly shaped clusters were excluded.

### Variables of the study

#### Dependent variable

Women’s health service access.

##### Women

In this study, women are all eligible women aged 15–49 years who are a permanent resident or visitors of the selected households available before the survey interview begin.

Women’s health services access was challenged by different factors. In this study, different factors that challenge women’s health services access were adapted from the EDHS report and other similar studies.^13^ Accordingly, women’s health service access was assessed regardless of the following four factors such as (1) not wanting to go alone, (2) distance to health facilities, (3) getting the money needed for treatment and (4) getting permission to go for medical care. Hence, the women had health service access if they had not been faced with any of the mentioned factors. Otherwise, the women had problems with health service access if they faced by at least one of the mentioned factors.

#### Independent variables

Age, educational status, wealth status, religion, media exposure and current working were used as individual-label independent variables. Region and place of residency were used as community-label variables.

##### Media exposure

Women’s health service access is related to their media exposure. Therefore, if the women had either radio, television or both the women had media exposure, and if the women had not had either radio or television, the women had no media exposure.^23^

### Data management and processing

Data cleaning, labelling and processing were done by using STATA V.15 software and Microsoft Office Excel. To yield accurate parameters estimation, and to handle the representativeness of the survey, sample weight was done.

### Statically data analysis

STATA V.15 software was used for data processing and analysis. A descriptive analysis was done to describe the characteristics of the study subjects.

### Spatial data analysis

ArcMap V.10.7 software was used for spatial autocorrelation and hot spot analysis. Global spatial autocorrelation (Global Moran’s I) statistic measure was used to assess whether women’s health service access was dispersed, clustered or randomly distributed. Moran’s I value close to −1, +1 and 0 indicates a dispersed, clustered and random distribution of health service access, respectively.^24^ Hot spot analysis (Getis-Ord Gi*) was done to know whether the women’s health service access is a hot or cold spot. The hot and cold spot values for spatial clusters were determined by z-scores and p values.

### Spatial interpolation

We used the ordinary Kriging interpolation technique to predict health service access in the unsampled EAs. Health service access in the unsampled EAs was predicted by interpolating the currently sampled areas.

### Spatial scan statistics

SaTScan V.9.5 software was used for the local cluster detection. Purely spatial Bernoulli-based models were employed to determine statistically significant clusters with high rates of women’s health service access.^25^ The women who were not faced health service access problems were taken as cases and those who had health service access problems were taken as controls to fit the purely spatial Bernoulli model. The default maximum spatial cluster size of less than 50% of the population was used as an upper limit, and to allow small and large clusters to be detected, and to ignore the clusters that exist the outside the maximum limits of the circular shape of the window. A log-likelihood ratio (LLR) test statistic was used to determine whether the number of observed cases within a cluster was significantly higher than expected or not. The circle with a maximum LLR was defined as the most likely (a primary) cluster. Then, all the remaining significant clusters were ranked based on their LLR.^26^ All most likely significant clusters were identified using p values, and ranked by their LLR test based on the 9999 Monte Carlo replications.^26^

### Multilevel mixed effect logistic regression analysis

Since the EDHS data had a hierarchical nature. Hence, women from the same cluster are more similar as compared with women who were from different clusters. Such kind of hierarchy of data might have a dependency nature. Therefore, this may violate the independence of observations and the equal variance of assumption. To overcome this violation, multilevel mixed-effect logistic regression models were assumed, and four models were considered to overcome if there is any data dependency: model 1 (a null model), model 2 (contains individual-level variables), model 3 (contains community-level variables) and model 4 (individual and community-level variables). For each model, the intraclass correlation coefficient (ICC) and variance were calculated to check the presence of data dependency and to apply multilevel mixed-effect logistic regression. ICC is used to the diagnosed correlation between clusters, and there are data correlation if ICC’s value is greater than 25%. Consequently, 40% of the ICC’s values confirmed that there was a significant correlation among women regarding their response to health service access. The LLR was used for model comparison, and the model with the highest LLR value was chosen as the best-fit model.^27^ As a result, model D was chosen as the best-fit model due to its LLR score’s highest value (−2598.4) as compared with other models (table 3). In addition, therefore, a multilevel mixed effect logistic regression analysis was fitted. A p<0.05 and a 95% CI were used to identify associated factors.

## Results

### Sociodemographic characteristics of the study participants

A total of 15 295 women (weighted) were included. More than one-third (36.7%) of women were from the Oromia region. The majority (77.8%) of women were rural residents. Nearest to half (48.2%) of women had no formal education. Two out of 10 women (21.3%) were under 15–19 years of age. Forty-six per cent of women were rich. Four out of 10 women (42.8%) were orthodox religious flowers. The majority (66.6%) of the women were not employed (table 1).

**Table 1:** Sociodemographic characteristics of the study participants

Variable	Category	Frequency (n)	%
Educational status of mother/caregiver	No formal education	7379	48.2
	Primary	5367	35.1
	Secondary	1721	11.3
	Higher	828	5.4
Region	Tigray	1099	7.2
	Afar	126	0.8
	Amhara	3533	23.1
	Oromia	5613	36.7
	Somali	457	3.0
	Benishangul	158	1.0
	SNNPR	3245	21.2
	Gambela	43	0.3
	Harari	38	0.2
	Addis Adaba	896	5.9
	Dire Dawa	88	0.6
Respondents’ age (year)	15–19	3259	21.3
	20–24	2655	17.4
	25–29	2893	18.9
	30–34	2299	15.0
	35–39	1911	12.5
	40–44	1278	8.4
	45–49	1002	6.6
Family’s wealth index	Poor	5339	34.9
	Middle	2914	19.0
	Rich	7043	46.0
Mother/caregiver religion	Orthodox	6545	42.8
	Catholic	120	0.8
	Protestant	3624	23.7
	Muslin	4797	31.4
	Traditional	123	0.8
Place of residency	Urban	3389	22.2
	Rural	11 906	77.8
Currently working	No	10 187	66.6
	Yes	5108	33.4

SNNPR, South Nations Nationalities and People's Region.

### Spatial distribution of women’s health service access

**Figure 1:**
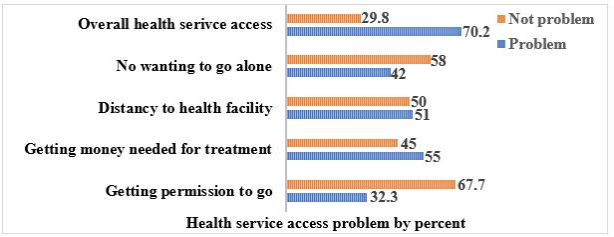
Health service access problem in Ethiopia, 2016 EDHS. EDHS, Ethiopian Demographic and Health Survey.

The women were assessed whether they had problems regarding health service access or not. Accordingly, women had a problem with not wanting to go alone (42%), distance to health facilities (51%), getting the money needed for treatment (55%) and getting permission to go for medical care (32.3%). Overall, 70.2% of women had at least one of the mentioned problems for health service access in Ethiopia (figure 1).

**Figure 2:**
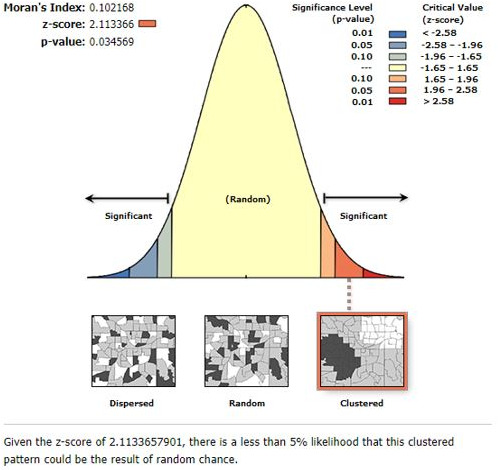
Spatial autocorrelation report of health service access in Ethiopia, 2016 EDHS. EDHS=Ethiopian Demographic and Health Survey.

**Figure 3:**
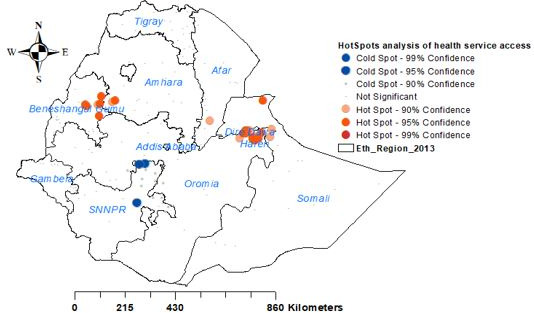
Hot spot analysis for health service access in Ethiopia, 2016 EDHS. EDHS, Ethiopian Demographic and Health Survey; SNNPR, South Nations Nationalities and People's Region.

The spatial autocorrelation revealed that the spatial distribution of health service access in Ethiopia was clustered (Global Moran’s I=0.102168, p=0.034569). These hot spots of health service access were observed in eastern Benishangul Gumuz, western Amhara, southern Afar, DireDawa, Harari and northern Somali regions (figures 2 and 3).

### Spatial SaTScan analysis

**Figure 4:**
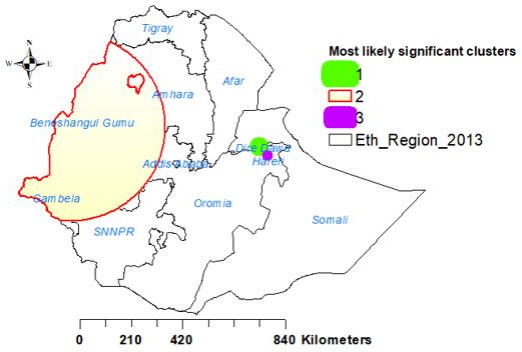
SaTScan analysis of health service access in Ethiopia, 2016 EDHS. EDHS, Ethiopian Demographic and Health Survey.

A total of 72 significant clusters were identified. Of these, 9, 62 and 1 cluster were primary, secondary and tertiary clusters, respectively. The primary and secondary clusters were located at 9.614701N, 41.829121E within a 5.69 km radius in Dire Dawa, and at 10.333829 N, 34.842459 E within a 386.61 km radius in Gambela, Benishangul-Gumuz, western Oromia and southwestern Amhara regions, respectively. Women who lived in the primary, and secondary clusters were 96% (RR=1.96, p<0.0001), and 39% (RR=1.39, p<0.0001) more likely to access health service than women who lived outside the window (table 2, figure 4).

**Table 2:** SaTScan analysis report of significant clusters for women’s health service access in the detected window in Ethiopia, using 2016 EDHS data

Types of cluster	Detected cluster	Coordinates/radius	Populations	Case	RR	LLR	P value
Primary	282, 285, 287, 286, 297, 296, 292, 290, 293	(9.614701N,41.829121E) 5.69 km	318	198	1.96	58.59	<0.001
Secondary	149, 151, 153, 152, 147, 154, 155, 160, 158, 170, 161, 159, 169, 167, 148, 168, 164, 163, 118, 162, 92, 80, 120, 79, 218, 211, 208, 94, 230, 229, 217, 220, 98, 53, 213, 52, 214, 206, 54, 81, 76, 97, 70, 59, 225, 85, 226, 221, 223, 210, 57, 71, 84, 96, 73, 99, 91, 95, 195, 201, 200, 112	(10.333829N,34.842459E)/ 386.61 km	1732	723	1.39	37.67	<0.001
Tertiary	247	(9.287253N, 42.135531 E)/0 km	26	21	2.43	12.39	<0.001

EDHS, Ethiopian Demographic and Health Survey; LLR, log-likelihood ratio; RR, relative risk.

### Interpolation of women’s health service access

**Figure 5:**
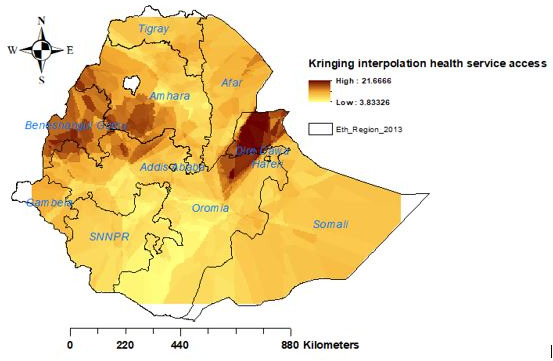
Ordinary kriging interpolation of health service access in Ethiopia, 2016 EDHS. EDHS, Ethiopian Demographic and Health Survey.

The kriging interpolation of women’s health service access revealed that there would be good health service access among women in Benishangul-Gumuz, western Amhara, Dire Dawa, eastern Oromia and northern Somali regions of Ethiopia. Whereas, women in the remaining parts of Ethiopia would face problems with health service access (figure 5).

### Factors associated with women’s health service access

In multilevel mixed-effect logistic regression analysis; education, wealth status, media exposure and residency were significant factors for women’s health service access.

The women who were in secondary, and higher education were 1.6 (adjusted odds ratio (AOR): 1.56, 95% CI 1.34 to 1.81), and 2 (AOR 2.02, 95% CI 1.66 to 2.44) times more likely to access health services than women who had no formal education. Rich women were 1.4 (AOR 1.38, 95% CI 1.19 to 1.61) times more likely to access health services than poor women. The women who had media exposure were 1.2 (AOR 1.15, 95% CI 1.03 to 1.29) times more likely to access health services than their counterparts. Rural women were 82% (AOR 0.18, 95% CI 0.14 to 0.23) less likely to access health services than urban resident women (table 3).

**Table 3:** Multilevel mixed-effect logistic regression analysis of women’s health service access using 2016 EDHS data

Variables	Category	Model 1	Model 2	Model 3	Model 4
			AOR (95% CI)	AOR (95% CI)	AOR (95% CI)
Educational status	Primary		1.19 (1.06 to 1.33)*	-	1.19 (1.07 to 1.34)
	Secondary		1.59 (1.36 to 1.85)*		1.56 (1.34 to 1.81)**
	Higher		2.10 (1.74 to 2.55)*		2.02 (1.66 to 2.44)**
	No education		1		1
Respondent’s age	20–24 years		1.04 (0.91 to 1.18)	–	1.03 (0.91 to 1.17)
	25–29 years		1.01 (0.88 to 1.15)	–	1.00 (0.88 to 1.14)
	30–34 years		1.06 (0.92 to 1.23)	–	1.06 (0.92 to 1.23)
	35–39 years		0.96 (0.83 to 1.13)		0.96 (0.83 to 1.12)
	40–44 years		0.95 (0.80 to 1.13)		0.94 (0.79 to 1.12)
	45–49 years		0.86 (0.71 to 1.04)		0.85 (0.70 to 1.03)
	15–19 years		1		1
Wealth status	Rich		1.35 (1.17 to 1.58)*	–	1.38 (1.19 to 1.61)**
	Middle		2.37 (2.04 to 2.74)*	–	2.26 (1.93 to 2.62)
	Poor		1		1
Respondents currently working	Yes		0.98 (0.91 to 1.09)		0.96 (0.90 to 1.09)
	No		1		1
Media exposure	Yes		1.16 (1.04 to 1.30)*	–	1.15 (1.03 to 1.29)**
	No		1		1
Region	Afar		–	0.64 (0.42 to 0.97)*	0.97 (0.65 to 1.45)
	Amhara		–	1.49 (1.03 to 2.17)	1.56 (1.10 to 2.22)
	Oromia		–	0.30 (0.21 to 0.45)	0.28 (0.19 to 0.40)
	Somali		–	0.58 (0.40 to 0.85)*	0.92 (0.64 to 1.35)
	Benishangul		–	0.52 (0.34 to 0.80)	0.56 (0.38 to 0.83)
	SNNPR		–	0.56 (0.38 to 0.82)*	0.53 (0.37 to 0.76)
	Gambela		–	0.67 (0.44 to 1.01)	0.78 (0.52 to 1.14)
	Harari		–	2.77 (1.79 to 4.29)	2.51 (1.66 to 3.79)
	Addis Abeba		–	0.89 (0.59 to 1.37)	0.66 (0.44 to 0.98)
	Dire Dawa		–	0.25 (0.16 to 0.39)*	0.25 (0.16 to 0.38)
	Tigray			1	1
Residency	Rural	–	–	0.19 (0.15 to 0.24)*	0.18 (0.14 to 0.23)**
	Urban			1	1
Model comparison	ICC	0.40	0.28	0.23	0.19
	Variation	0.17	0.105	0.081	0.072
	MOR (95% CI)	2.22 (1.92 to 2.57)	1.29 (1.10 to 1.52)	0.97 (0.82 to 1.15)	0.81 (0.68 to 0.97)
	AIC	16 866	16 218	16 502	16 027

*Significant at model 2 and model 3; **, significant at model 4.

AIC, Akaike's information criteria; AOR, adjusted odds ratio; CI, confidence interval; EDHS, Ethiopian demographic and health survey; ICC, intraclass correlation coefficient; MOR, median odds ratio; SNNPR, South Nations Nationalities and People's Region.

## Discussion

For this study, 2016 EDHS data were used that was accessed from the DHS website. Through request, permission was obtained to access the data. There are no attributes that uniquely identify individuals’ women or household addresses in the data files. This is because the geographical coordinate files are randomly displaced within a large geographical area, and it is only for EAs as a whole. As a result, specific enumeration areas (ERs), individuals’ women and households cannot be identified uniquely. The shape file of Ethiopia was taken from the open Africa website. A two-stage stratified cluster sampling technique was used, and all women under the age of 15–49 years were the study population. Since the data have hierarchical nature, data dependency might have existed. Therefore, ICC was used to assess data dependency. Based on the result, multilevel mixed-effect logistic regression models were considered to alleviate the data dependency, and different model selection criteria were assumed to select the best-fit model. For spatial analysis, spatial autocorrelation and hot spot analysis were used to assess the distribution of data, and identify the hot or cold spot areas of women’s health service access, respectively. The ordinary Kriging interpolation technique and purely spatial Bernoulli model were used to predict unsampled areas, and to detect local clusters of women’s health service access, respectively.

Women’s health service access was assessed to determine whether they had problems regarding health service access or not. Accordingly, respondents had problems getting the money needed for treatment (55%), distance to health facilities (51%), not wanting to go alone (42%) and getting permission to go for medical care (32.3%). Overall, 70.2% of women had at least 1 of the mentioned problems with health services access, and only 18.9% of women had good health service access in Ethiopia. This evidence was supported by studies done in Nigeria^11^ and Ethiopia.^28^ This finding was also supported by women’s suboptimal ANC visits in Ethiopia, which ranged from 10.0% to 32%,^29^ and low utilisation of PNC service utilisation.^9^ This might be because women living far from health facilities are less likely to use or access healthcare services, their poor perception of the available healthcare services and lack of transportation services. In addition, mothers might not know about signs of pregnancy complications, women’s low health-seeking behaviours, inaccessibility of health institutions and women’s low ANC and PNC visits.^11^ Therefore, stakeholders create awareness for women to make them volunteer to go alone for medical care, and husbands and other relatives might prevent women to go to the health facility and access the respective health service. So, awareness is also created for husbands and relatives not to prevent women to access health services. Furthermore, nearby health facility for women is critical to ensure equal health service access and to meet the target of maternal and child healthcare services utilisation. As well as policy-makers should enhance the economic status of women.

The spatial distribution of health service access in Ethiopia was not random. High health service access was observed in eastern Benishangul Gumuz, southwest Amhara, southern Afar, DireDawa, Harari and northern Somali regions. The primary and secondary clusters were located in Dire Dawa, Gambela, Benishangul-Gumuz, western Oromia and southwest Amhara regions, respectively. Women who lived in the primary, secondary and tertiary clusters were more likely to access health services. The Kriging interpolation of women’s health service access revealed that there would be good women’s health service access in Benishangul-Gumuz, western Amhara, Dire Dawa, eastern Oromia and northern Somali regions. This finding was supported by a similar study done about women’s home delivery that states a high proportion of home delivery is found in Amhara, Afar, Tigray, Oromia, and South Nations Nationalities and People's Region,^30^ and incomplete maternal continuum care utilisation.^13^ Therefore, policy-makers should give priority attention to the areas where women had less likely to access health services in Ethiopia.

In the multilevel mixed effect logistic regression analysis, secondary and higher educational status, rich wealth status, and exposure to media were positively associated, and being a rural resident was negatively associated with women’s health service access, respectively.

Women with secondary and higher education were 1.6 and 2 times more likely to access health services. The current evidence was similar to studies done in Ethiopia^17 18^ and the Republic of Vanuatu.^31^ This might be education’s power to enhance women’s health-seeking behaviours, educated women actively involved in reading materials and discussions that would enhance their knowledge.^17^ Moreover, educated women might give priority attention to their health, strive to know the benefits of healthcare services and illiterate women may fail to receive health services during pregnancy.^32^ In line with this finding, stakeholders should enhance women’s educational status by using different educational delivery mechanisms, for instance, a health professional could provide appropriate consultation service during women’s health facility visits, and educational messages for women could be sent to women through short message services. Rich women were 1.4 times more likely to access health services. This finding was similar to studies done in Ethiopia^17 18^ and the Republic of Vanuatu.^31^ This might be women’s better economic status which increases their healthcare-seeking behaviour and autonomy in healthcare decision-making, they may afford to cover medical and transportation costs. Furthermore, wealthy women may cover their drug and transportation costs.^18^ In addition, poor women could have poor utilisation of preventive, promotive and curative aspects of health services. So, policy-makers should enhance women’s wealth status, and encourage women to have their daily income.

The women who had media exposure were 1.2 times more likely to access health services. This finding was similar to studies done in Ethiopia^13 18^ and Nepal.^33^ This could be the power of mass media in disseminating information concerning maternal health that may enhance women’s knowledge and attitude towards health service access and utilisation.^33^ Furthermore, women who were exposed to the media were more likely to be informed about health services utilisation. Therefore, the availability of media spots is critical to the delivery of health-related information messages that could reach out to women in their homes.

Rural resident women were 82% less likely to access health services. This finding was similar to studies done in Ethiopia.^17 18^ This might be because health facilities are inadequately accessible and available in rural areas. Rural resident women might be limited in access to education and health information.^27^ Moreover, in rural areas adequate health professionals might not be available and so appropriate counselling services might not be delivered. In resource-limited settings, health facilities and necessary infrastructure such as roads and clean water are less likely available in the rural side of the country. Therefore, stakeholders better if they close such gaps in the rural areas to ensure women’s health service access and utilisation.

## Conclusions

In Ethiopia, inadequate numbers of women had good health service access. Women had faced problems with getting money for treatment, distance to health facilities, not wanting to go alone to health facilities and getting permission to go for medical care. The distribution of women’s health service access was spatially clustered in Ethiopia. Women’s health services access was positively associated with education, wealth and media exposure. However, rural resident women were negatively correlated with health service access. Therefore, stakeholders should pay priority attention to the cold spot areas of women’s health service access. Improving women’s educational status, and providing women with various media access, is critical for health service access. In addition, stakeholders should create job opportunities for poor women, and deliver public health promotion regarding maternal and child health service utilisation. Providing appropriate consolation services and constructing nearby health facilities are also possible interventions to enhance women’s health service access.

### Strengths and limitations

This study analysed nationally representative data and a multilevel logistic regression analysis model that could alleviate data correlations were employed. Appropriate health intervention techniques that would increase women’s health services access were highlighted. Since the study was based on cross-sectional, social desirability and recall bias might exist. So, the finding might have a temporal relationship. Moreover, the coordinate file was not originally collected at the four-corner direction of Ethiopia. So, this study excludes areas that had no coordination file (at four corners of Ethiopia), and irregularly shaped clusters that were not detected in the SaTScan analysis were excluded.

## Data Availability

All data relevant to the study are included in the article or uploaded as online supplemental information.
